# Exploration of Infertile Couples’ Support Requirements: A Qualitative Study 

**DOI:** 10.22074/ijfs.2015.4212

**Published:** 2015-04-21

**Authors:** Fatemeh Jafarzadeh-Kenarsari, Ataollah Ghahiri, Mojtaba Habibi, Ali Zargham-Boroujeni

**Affiliations:** 1 Nursing and Midwifery Student Research Center, Faculty of Nursing and Midwifery, Isfahan University of Medical Sciences, Isfahan, Iran; 2 Department of Obstetrics and Gynecology, School of Medicine, Isfahan University of Medical Sciences, Isfahan, Iran; 3 Family Research Institute, Shahid-Beheshti University, Tehran, Iran; 4 Nursing and Midwifery Care Research Center, Faculty of Nursing and Midwifery, Isfahan University of Medical Sciences, Isfahan, Iran

**Keywords:** Crisis, Infertility, Needs, Qualitative Research, Support

## Abstract

**Background:**

Due to high prevalence of infertility, increasing demand for infertility treatment, and provision of high quality of fertility care, it is necessary for healthcare professionals to explore infertile couples’ expectations and needs. Identification of these needs can be a prerequisite to plan the effective supportive interventions. The current study was, therefore, conducted in an attempt to explore and to understand infertile couples’ experiences and needs.

**Materials and Methods:**

This is a qualitative study based on a content analysis ap- proach. The participants included 26 infertile couples (17 men and 26 women) and 7 members of medical personnel (3 gynecologists and 4 midwives) as the key informants. The infertile couples were selected from patients attending public and private infertility treatment centers and private offices of infertility specialists in Isfahan and Rasht, Iran, during 2012-2013. They were selected through purposive sampling method with maximum variation. In-depth unstructured interviews and field notes were used for data gathering among infertile couples. The data from medical personnel was collected through semi-structured interviews. The interview data were analyzed using conventional content analysis method.

**Results:**

Data analysis revealed four main categories of infertile couples’ needs, including: i. Infertility and social support, ii. Infertility and financial support, iii. Infertility and spiritual support and iv. Infertility and informational support. The main theme of all these categories was assistance and support.

**Conclusion:**

The study showed that in addition to treatment and medical needs, infertile couples encounter various challenges in different emotional, psychosocial, communicative, cognitive, spiritual, and economic aspects that can affect various areas of their life and lead to new concerns, problems, and demands. Thus, addressing infertile couples’ needs and expectations alongside their medical treatments as well as provision of psychosocial services by development of patient-centered approaches and couple-based interventions can improve their quality of life and treatment results and also relieve their negative psychosocial consequences.

## Introduction

Infertility is a stressful experience and complicated crisis for infertile couples (1,2) which can coincide with various social, psychological, physical and financial stresses (3). Researchers have compared the stress induced by infertility with the feeling when losing a child or a spouse (4). Infertility was defined, in the International Conference of Infertility in Bangkok in 1998, as a global health problem with physical, psychological and social dimensions (5). The prevalence of infertility ranges from 5 to 30% in different countries (6). The prevalence of primary infertility has been reported as 24.9% among Iranians in 2004. In addition, about a fourth of Iranian couples have experienced primary infertility at some point in their lives (7). 

Inability to achieve pregnancy is usually coupled with low confidence, depression, sexual problems, feeling of shame or guilt, lack of communication with friends and family members and occupational challenges. These conditions can be deteriorated in different socio-cultural contexts, especially when being concurrent with medical, emotional and communicative challenges triggered by infertility (8). Infertility can negatively affect social, personal and marital relations that results in a mental imbalance leading to divorce (6). The socio-cultural context can be an important factor in one’s conceptualization of and response to, infertility (9). 

Nowadays, there are tremendous changes in family values, but parenthood has still been an undeniable importance for women and men (10). Infertility carries much social stigma in many communities, leading to many social problems for infertile couples (11). In Iranian culture, infertility coincides with numerous psychological and social challenges, especially for women, and affects all aspects of infertile couples’ life (10,12), including their emotional, psychological, physical and social performances (11). 

Diagnosis and treatment of infertility is usually coupled with noticeable stress (13). Assisted reproductive technology (ART) has been regarded as one of the most stressful infertility treatment method. The application of these technologies is coupled with emotional and physical burden and a high level of depression and stress (1,14,15). Thus, infertility and its treatment are considered as significant medical issues affecting quality of life of infertile couples (11,16). 

Various studies have demonstrated that infertile couples have a variety of needs (12,15,17,20) that require emotional support, professional psychosocial services, couple-centered interventions and patient-centered approaches (21). Due to the diversity of needs and problems of infertile patients, this necessitates to identify and address infertile patients’ needs along with their regular medical treatments. Provision of fertility care based on patients’ needs and expectations (patient-centered fertility care) is considered as one of their fundamental right (22). Patient-centered fertility care not only fulfills patients’ needs and expectations, but also has significant clinical benefits such as improving patients’ quality of life and emotional health as well as decreasing their distress (12,15). In addition, it is recommended to provide infertility treatment alongside patient-centered care in order to promote patients’ well being during treatment and increase treatment success rates (23). To this end, healthcare professionals are in a good position to sympathize with infertile patients during their treatment period and encourage them to express their experiences, needs and concerns (24). 

Different aspects of infertility have been studied in various quantitative research works in Iran. However, qualitative studies are so limited and the nature of Iranian infertile couples’ needs has not been completely justified yet. Exploration of infertile couples’ viewpoints on their needs can be a prerequisite for planning effective supportive treatment interventions (3,24,25) for fulfilling these needs and improving treatment capabilities of healthcare professionals. On one hand, need is a complicated phenomenon and is rooted in the cultural, social and economic context of communities (26). On the other hand, quantitative approaches lack the required capability for providing a detailed description of the phenomena and patients’ viewpoints. Thus, qualitative researches seem to be appropriate for exploration of needs and experiences regarding fertility care (15). They also offer exclusive capability to provide a deeper understanding of phenomena (27). In addition, they can provide descriptions of life experiences, human interpretations and perceptions in the respective cultural and social contexts (27,28). Thus, considering the existing gap, the current qualitative study has been conducted in an attempt to explore and understand infertile couples’ experiences and viewpoints with regard to their needs. 

## Materials and Methods

### Participants and data gathering methods

This study was part of a larger sequential exploratory mixed methods research with the purpose of identifying infertile couples’ needs, development and validation of a tool for measuring couples’ needs. The first phase of this mixed methods research was a qualitative study with a content analysis approach. The main purpose of this qualitative phase was exploration of infertile couples’ needs and identifying the primary items of the needs assessment tool. The second phase of the research was a quantitative study aimed at validation of the needs assessment tool. Part of the data obtained from qualitative interviews with infertile couples and key informants was reported in this paper. Another part of the data obtained from qualitative interviews with 17 infertile couples was reported in another published paper (22). 

This study was conducted in settings and places, like a state-run infertility center in an university hospital, private offices of infertility specialists and private infertility centers in Isfahan and Rasht, Iran, during 2012-2013, where infertile couples were accessible. Due to insignificant of the study population in qualitative research projects (29), there was no limitation for places as research setting for the current study. Participants of the study included 26 infertile couples. 

The researchers interviewed each infertile couple using a couple-based approach. This approach is appropriate because infertility is a condition involving the two partners in a joint relationship (30). A dyadic approach were applied for 17 couples to establish a better interaction between spouses and interviewer, while to provide a clearer picture of couples’ needs and viewpoints. Due to husbands’ busy schedules (n=3), their unwillingness to participate in the interviews (n=4), becoming ill at the time of interview (n=2) and only wives (n=9) as representatives of the couples were individually interviewed. 

In addition, interviews with seven medical professionals (3 gynecologists and 4 midwives) as key informants were carried out to obtain more comprehensive and deep information regarding infertile couples’ needs that may be overlooked by couples. Interviews were carried out in infertility care centers or the private offices. 

Infertile couples were selected through purposive sampling method with maximum variation, including different causes of infertility, different types of infertility (primary and secondary), a wide range of age, at different stages of infertility treatment and different durations of infertility. The inclusion criteria for infertile couples were as follows: at least one year after marriage, confirmation of their infertility by the specialist, Iranian nationality, proficiency in Farsi, willingness to participate in the study, no history of mental disorders (during last 12 months) or physical disabilities and ability to express their feelings and experiences. Also the medical personnel were selected through purposive sampling method and their inclusion criteria were as follows: willingness to participate in the study, adequate experience in infertility treatment, and caring for infertile couples. All participants were interviewed in one or two sessions lasting approximately 20 to 60 minutes. 

In-depth, unstructured interviews and field notes were used for data gathering among infertile couples. The general question of the research posed for the infertile couples was "Tell me about your infertility experiences and the problems (concerns) you have encountered in this regard." In proportion to the received answers, explorative questions were asked for expansion of findings such as, "Can you mention some more examples? and What does that mean?". Demographic information were also asked in addition to questions related to the experience of infertility, such as: age, gender, education, causes of infertility and its duration, number of children in couples with secondary infertility, type of treatments received, etc. Researchers also used field note-taking for data gathering to this end, the first author attended the infertility treatment clinics and infertility specialists’ offices so as to observe the interaction between patients, their families and medical personnel. 

Semi-structured interviews with medical personnel were carried using questions extracted from main subjects and ideas mentioned by infertile couples in their interviews. Some of the questions asked during interview sessions with the medical personnel were the following: "In your opinion, what issues and problems are infertile couples facing?", "What problems are these couples facing during diagnosis and treatment procedures?", "What are their most significant needs?", "What solutions do you recommend for fulfilling their needs?", "Is the quality of behavior of medical team important for motivating patients to continue their treatment process?", "Are patients usually complaining about the presence of other patients in the office during their check-up time?", and "What is your idea on this?". 

Interviews and field note-takings were continued up to data saturation. All interviews were conducted in Farsi by the first author and then translated into English. All interviews were recorded, transcribed verbatim, and then analyzed concurrently. The interview data were analyzed using conventional content analysis. 

### Data analysis

Drawing on steps recommended by Graneheim and Lundman (31), the following procedure was employed for analysis of the collected data: i.Transcribing all interviews immediately after each interview, ii. Reading the whole transcription for general comprehension of the content, iii. Determining the number of meaning units and primary codes, iv. Categorizing similar primary codes in more comprehensive categories and v. Determining the latent content (themes) in the data. 

### Trustworthiness of data

To ensure the credibility, feedback was obtained from participants (member checking) and the number of interviews with some participants was increased. To increase the transferability of findings to other settings and groups, participants with various experiences in the realm of the subject under study and with maximum variation were selected. Also, confirmability and dependability of findings were established through peer checking, peer debriefing, reviewing of transcripts by some participants, researchers’ interest in the phenomenon under study and their prolonged engagement in data. 

### Ethical considerations

This study were confirmed by the Ethical Committee of Isfahan Universtiy of Medical Sciences, Isfahan, Iran. Also, prior to interviews, participants were made aware of the objectives of the research and an informed consent was obtained. Oral and written permissions were obtained from them for recording their interviews and they were assured that the gathered data would only be used for research objectives. It was also announced to the participants that they could withdraw the research anytime they wish and their information would remain confidential during and after the research. 

## Results

Out of 26 couples (n=43) participating in the study, females’ age ranged 20 to 47 with a mean age of 31.36, while males’ age ranged 25 to 55 with a mean age of 36.5. Out of 7 members of medical personnel, two were female, one was a male gynecologist and four were midwives. Twenty couples (76.9%) had primary infertility and the rest experienced the secondary type. 

Duration of the couples’ infertility ranged 1 to 21 years (Mean: 4.44 years) and their treatment ranged 1 month to 20 years. Infertility was of female-related in 15 couples, of male-related in 5 couples and of mixed causes in 4 couples. Moreover, two couples had unexplained infertility. All couples with secondary infertility, except one who had no children (with a history of still birth in 28^th^ week of the first pregnancy in 3 years ago), had one child. Educational level of female and male participants ranged from secondary school to higher education (university).

Data analysis yielded four main categories of infertile couples’ needs including: i. Infertility and social support, ii. Infertility and financial support, iii. Infertility and spiritual support and iv. Infertility and informational support. Assistance and support constituted the main theme of all the categories (Table 1). 

**Table 1:** Themes, categories and sub-categories


Theme	Categories	Sub-categories	

**A need for assistance and support**	Infertility and social support	-Spouse’s support	
		-Familial and social support	
	Infertility and financial support	-Efficient medical insurance	
		-Support from authorities of governmentaland non-governmental agencies	
	Infertility and spiritual support	-Hope in God	
		-Communication with God	
	Infertility and informational support	-Need to information on disease	
		- Need for educating and preparing thefamily and society	


## Infertility and social support

### Spouse’s support

A demand for mutual understanding between husband and wife, their emotional support for one another, the compassion and love shared between them, encouraging persistence with treatment, accompanying each other during the treatment and respect for spouse’s opinion about the method of treatment were among the instances emphasized by participants in their interviews. It can be inferred from the most participants’ statements that spouse’s full support can be encouraging and can inspire self-confidence, security and equanimity in the onerous path of infertility experience. One of the women (couple number: 15) stated in this regard:

I was so upset when I found out about my problem. I felt so desperate and completely hopeless. I really needed my husband’s support. Fortunately, my husband was there by my side supporting me.

This woman’s husband added:

I didn’t want her to think that I am pretending in front of her. In our marriage the most important things for me are her health and peace of mind. I have already told her many times and am telling her now, that we have no problem. She should not get disappointed. This is surely God’s will not to have any kids for now.

As the burden of infertility falls principally on women, they are mainly involved in infertility diagnosis and treatment processes; therefore, female participants showed a greater need for the spouse’s accompaniment during the treatment process. Most of female participants preferred their spouse by their side when attending infertility clinics. One of the participants (couple number: 1) stated in this regard:

I like to have my husband by my side to empathize and support me when coming to clinic, but men do not like to sit somewhere idle, they are very impatient and always in rush to get back to work.

This woman’s husband added:

She is right, it is difficult to be alone. I know that she needs my support, but I am so busy and not able to leave my office to accompany her for coming to the clinic in the morning. When I have to be here, I would do my best. But she must to understand my work condition.

But some of the female participants had no tendency for having their husband by their side during the whole process of treatment. One of the participants (woman number: 24) said in this regard:

I would like my husband to be with me in some places. But also I prefer not to involve him in some certain places. Sometimes I think I can decide better when I am alone. Because I think it is my problem. It is better not to inform him initially. I will break the news to him little by little and I believe this makes it easier to get his acceptance. Hopefully, he will response positively.

"They don’t let husbands into the doctor’s office as they send three women together and they say that men are 'namahram' (i.e. men or women other than one’s blood relatives and spouse). Islam bans any relationship with a Namahram person that involves seeing any part of the body except the face, hands and feet. Therefore, I didn’t come with my wife anymore." one of the men (couple number: 10) said and tried to explain why he did not accompany his wife to gynecologist’s office. Also this man’s wife added: 

Anyhow, doctor’s office is far away from where we live, so it gets dark by the time we get back home. As he said, he refuses to accompany me, so I had to ask my sister to be with me to avoid being alone. 

One of the gynecologists participating in the study said: 

This is one of the major problems we are all facing in this regard. This is due to overloaded doctors’ office. We are not like developed countries where the government mandates that only 4 patients must be visited in an hour. If I visit four patients in an hour, I would be at my office until next day. Therefore, I must visit patients in group of four per office visit. 

Another gynecologist stated in this regard: "I believe this is necessary and this is considered as patient’s right. Scientifically speaking, both men and women need to be visited in their first referral. But this is impossible due to overloaded doctor’s office." Another participant (couple number: 4) mentioned why her husband refused to accompany her to the gynecologist’s office, although she admitted that she didn't like him to do so: 

My husband supports me financially during the treatment, but he prefers not to be with me during the visits. He thinks that this is a women issue to handle and I must do it by myself. 

This woman’s husband added: 

A gynecologists’ office is always full of patients, and there is not enough room to sit, even for pregnant women. To be honest, men should not be there. 

Although most of the participating couples asserted that infertility had no negative effect on their interpersonal relations with their partner, they believed their attempts for having a baby were to ease the loneliness they felt, to preserve the sweetness of their life, to strengthen their relationship and to value loyalty and faithfulness of their spouses. This was more evident in infertile women as they saw the continuation of infertility and a childless life as a threat to their marital life and a trigger for separation and divorce. One of the participants (couple number: 5) said: "I had always the feeling of fear that my husband would decide to marry another woman because I couldn’t get pregnant. I used to have this fear". This woman’s husband said: 

I don’t know how these thoughts came to her mind. I have told her several times that I love my life. I believe that, it’s God’s will to have a kid or not. 

Some of the female participants (couples numbers: 12, 14, 15, and 17) also believed that if the infertility problem could not be treated and they would have no children of their own, the husbands should understand their ever-greater needs for pregnancy, child bearing or having children for easing their loneliness. They also should cooperate and agree on alternative solutions such as donated embryo or adoption. In this regard, some of husbands disagreed with oocyte or egg donation and adoption. Some also chose adoption over egg donation. Some also said that they had not thought about these and had no idea. Some women (women numbers: 8, 20 and 22) also were not willing to adopt any kids or receive any donated oocyte or embryo. One of the gynecologists said: 

Something must be done in this regard, so receiving embryo and oocyte of someone else become more understandable. One of the current problems is that assisted reproductive methods such as egg donation or gestational surrogacy are not culturally accepted for infertile couples or their families. 

### Familial and social support

Other important points in participants’ experiences were as follows: i. Sympathy and emotional support provided by their family, friends, and society, ii. Respect for couples’ privacy, iii. Encouragement for continuation of treatment, iv. Offering hope, v. Social acceptance and vi. Vocational support for the employed infertile couples. Due to judgment made by others and families, especially those of husband’s, their direct and indirect interventions, the social stigma attached to infertile patients, discouragement and hopelessness in continuing the treatment, most of the patients (who were mainly women) tried to restrict their social relations and tell white lies in response to others’ curiosity with regard to their infertility."Currently, my husband and I have tried to limit our relationship with his family for this issue, once in every two or three months." one of the couples (couple number: 20) said in this regard. One of the male participants (couple number: 11) stated: 

Others used to ask me whether we had problem to get pregnant, but I reply that there was no problem and we did not want to have any kids. As we are living independently, we don’t let others interfere with our privacy to cause further problems. 

Two of female participants (couples numbers: 16, 23) whose infertility was male-related stated that they preferred to let their husband’s family know about the cause of the problem to remove any doubts for infertility. One of the male participants (couple number: 10) who had an interesting opinion stated: 

Our families know that our problem is female related infertility. I wanted them to know the true cause of the problem. Otherwise, they would doubt me and think it’s my fault. I didn’t want them to have such an idea about me. 

"I don’t know. It was his idea and I had to tell our families the truth. Although it was a bit difficult for me, my husband wants the other do not blame him for infertility," this man’s wife mentioned. 

The life of infertile couples is affected considerably by reactions and behaviors of families and friends. Unnecessary interventions and some behaviors, like pity, made by families annoy the couples (especially women), disrupt their equanimity and often destabilize their relationships. "We expect family and acquaintances to show less pity and to refuse to say such things, ‘they have no kids, or our brother does not have any children yet,’ that is so annoying," one of participants (couples number: 13) mentioned. "I think family support is very important. Both families of the husband and wife should participate in this issue. They should not look for someone to blame," one of the participating gynecologists stated. 

Few couples also expressed their satisfaction and happiness with the supportive and positive role of their families in encouraging them to continue the treatment, maintain their equanimity during the treatment and bear the problems until achieving their goal. 

Some couples were annoyed by the behavior and negative approach toward infertility that exerted by the society. "I heard from one of my students’ parents saying that they should not enroll their kids in so-and-so’s class as she is nervous and impatient with kids (tearful eyes)," one of the participants (couple number: 8) stated in this respect. "It is not like this at all. Both my wife and I love children dearly. Although we do not have any children, it doesn’t mean that we treat people and their children badly," this woman’s husband added. 

As women are more involved in infertility treatment process, the employed women were concerned to leave the office in order to participate in treatment programs. "I am unable to leave the office, easily. This is also one source of stress and anxiety," one of the participants (woman number: 24) stated in this regard. 

## Infertility and financial support

Enormous expenses of infertility diagnosis and treatment, the long and iterative nature of these processes and patients’ financial limitations were among patients’ gravest concerns that were underlined frequently in their interviews. Most of the participants counted the enormous expenses and their financial problems as the cause of delayed commencement, or probable discontinuity of treatment procedures in the case of failed initial efforts. A demand for financial support from family and acquaintances, insurance companies, as well as cooperation from governmental authorities and nongovernmental entities were emphasized repeatedly in participants’ statements and field notes taken during the study. One of the couples (couple number: 9) in this regard mentioned: 

We mostly borrow some money for our treatment. We are not in a good financial condition. 

Nobody in our families could help us. In short, authorities should support more those who are in bad financial situation. 

One of the gynecologists participating in the study said: 

I think one of the major problems is their financial challenges. We witness patients who practically sell their houses and get large loans, so they can pay for infertility treatment expenses. 

Another couple (couple number: 3) said: "We wish infertility were treated like special diseases and would get the same degree of attention. We wish insurance would pay for the treatment and medical expenses." "Government should help and pay for part of treatment cost. It would be great if we could have an infertility committee like Emdad Committee." Another couple (couple number: 5) mentioned. 

## Infertility and spiritual support

A need for a superior power (God), faith in God’s will, connection with God and asking for His help were among other issues which were emphasized by most of participants. One of couples (couple number: 6) said in this regard: "We are sure that we are under supervision of God compassion. We have faith in God’s will. We are satisfied with His will and decision. Doctors are just a means that will help us." Another female participant (couple number: 7) added: 

I used to cry and be severely depressed, but praying gives me hope and helps to imagine myself having a kid. I ask God to help me to tolerate not having the child. Praying can lift me into a new lightness of heart. "I believe it is not late at all. My wife is not too old to have a kid. Anyhow, we have faith in God’s will. First God and then the doctor will help us. Nothing happens if God does not give us any kids," added by this woman’s husband. With regard to the significance of infertile couples’ religious and spiritual values, one of the midwives participating said: 

All people live with hope. Trusting God and prophets can help the patients a lot in their life. 

It might be better to have a religious counselor in infertility centers, so these couples could receive some religious consultation before and after treatment, so they may find some peace. 

Some of the participants believed that their infertility was a kind of divine trial and they should be satisfied with God’s will and should not be ungrateful as God knows their interest better. Some also viewed infertility as a divine punishment for their past ungratefulness. "I was not interested in kids when I was single. I sometimes think my husband’s infertility is a kind of punishment by God," one of the participants (woman number: 23) mentioned. "We tell ourselves we may have committed a sin that is why we have such problems. Perhaps God is testing us," another couple (couple number: 2) mentioned. 

## Infertility and informational support

The other issue emphasized in participants’ interviews was a demand for obtaining comprehensive information from the medical and treatment teams during diagnosis and treatment of infertility. Educating society with regard to infertility and new available treatments were other aspects of the issue emphasized by participants. Patients’ inadequate knowledge on the nature of the condition, the outcomes of a diagnostic and treatment method and ignorant behavior of medical teams to patients’ questions were mentioned by participants as the current problems in the treatment process. Educating and providing information to patients lead to their trust and cooperation during the treatment. "We are so unhappy because they refuse to explain what the problem is, what the cause is, how long the treatment takes, or how hopeful we can be on the success of treatment. We have to search the web to find some answers," one of the couples (couple number: 2) mentioned in this regard. Another couple (couple number: 3) said: 

If the doctor gives some information about our condition, we would worry less. Sometimes medical personnel answer some questions may cross our mind. But the answers are uttered too fast that we don’t understand a word they are saying. 

One of midwives participating in this study recommended as the following on the necessity of information dissemination and training on infertility: 

It may be better to have informative and training courses for the youth on the issue of infertility, its preventive methods and treatment in universities. It is not a bad idea to acquaint the youth with this phenomenon before marriage. 

One of the gynecologists participating in the study said: 

This must be cultivated in our culture. Educational movies should be made and broadcast in public media for expanding the general knowledge on this issue. People should know that, due to the scientific progress made recently, there is no such thing as infertility anymore, but there are solutions. 

## Discussion

The current study is the first qualitative study trying to explore the Iranian infertile couples’ needs. A close overview of the research findings from interviews showed that a need for support and assistance is among infertile couples’ main demands, so that they can cope with the stress caused by infertility. This need includes four main categories as follows: i. Infertility and social support, ii. Infertility and financial support, iii. Infertility and spiritual support and iv. Infertility and informational support. 

The findings of the study showed that spouse’s support is one of the main sources of support for patients, especially for female infertile patients. Even when family and acquaintances fail to play a supportive and positive role, spouse’s empathy, affection, loyalty and adequate support can provide the necessary emotional support for the partner to keep her/his hope alive and to be confidence in order to continue the treatment program. Results obtained by Akizuki and Kai’s study on Japanese infertile women suggested that partner’s support plays a vital role and decreases the need for others’ support (32). Abbasi-Shavazi et al. (33) realized that the infertile women who were supported by the good behavior of their husbands during the treatment processes, despite having no children, could manage their life and activities more efficiently. Infertile couples need each other’s support to better cope with their issue, so if one partner evades his/her responsibilities, the other partner who is usually the woman (not always) would be hurt (34). 

Another interesting finding in this study was attitudes expressed by most participants about spouse’s accompaniment during diagnosis and treatment of infertility. As mentioned in the results section, in Iran, like other developing countries, treatment of infertility is mainly taken up by women without taking the causes of infertility into consideration. This issue adds to their burden of responsibility and increases their physical and psychological stress (35). Thus, most female participants considered spouse’s accompaniment as an important source of support, and interpreted their spouse’s absences as: busyness, lack of enough space in clinics (especially in gynecologists’ offices), socio-cultural limitations, the great number of patients attending these centers and time limitations for patient visit. To encourage and improve men’s cooperation in infertility treatment programs, effective policies are required for removing cultural, religious, ethical and social barriers rooted in the society. Apart from basic facilities, persistence, more supervision by national health care authorities, adequate staff training, facilitating flexibility in clinics organization and educating the society are also required. 

Considering the significance of spouse’s support and its effect on the relationship between the couples as well as the stress induced by infertility (especially in women), infertility should be considered as an issue involving both men and women in clinical settings (36). Thus, healthcare professionals should consider an infertile couple as a unit and pave the way for husbands’ participation in diagnostic-treatment programs. These programs help husbands to change their views and interests and facilitate their cooperation during the treatment process and consequently improve the relationship between the wife and the husband (37). 

The other finding of the current study was couples’ various viewpoints on donated embryo and oocyte, gestational surrogacy, and adoption. This challenge is rooted in cultural and religious beliefs and attitudes of participants. This is in keeping with the findings obtained by previous studies conducted on this subject (12,38). 

The findings of the current study also showed the support from the family, acquaintances and society as the other important need, expressed by the infertile couples. Evidence has shown that positive social interactions and socio-emotional support have a salutary effect on infertile couples’ psychosomatic health, ultimately leading to a decrease in the negative impacts of stress. In addition, they psychologically adapt better and take a proper action against infertility and accept the situation more easily (4, 36, 39). Adequate social support and understanding from family and friends help infertile men and women feel better about themselves, establish a better relationship with others, and respond better to the treatment (4, 40). Evidence has shown that women more than men tend to have social support. The results of other studies have shown that family support affect infertility stress in women, directly and indirectly. Family support not only decreases social problems of infertility, but also leads indirectly to a decrease in infertility stress in four aspects of communicative problems, sexual problems, non-acceptance of a childfree lifestyle and a need for parenthood (36). It is also worth to note that people tend to have their family and friends’ support for adapting to the issue of infertility. Some others tend to hide their infertility and want to receive no support from others (41).

Due to the specific cultural and social structure in Iran, the issue of infertility takes a deeper meaning in the Iranian social context. Hence, the role of different tribes, acquaintances and friends proves to be significant and even vital, in infertile couples’ life (20). To this end, the findings of our study indicated that most infertile couples, especially infertile women, preferred to keep their infertility issue as a secret and avoid mentioning it, especially to their in-law’s family. This finding is consistent with those obtained by other studies (12, 20, 33). Mollaiy nezhad et al. (42), have mentioned that the responsibility of infertility is on women’s shoulder in most of the communities and her infertility is usually rebuked by her in-law family. This issue ultimately leads to the concern that woman’s infertility is an adequate cause for divorce and man’s marriage with another woman.

In addition, the current study showed that some of the participants had to tell the truth to their inlaw families due to financial dependence, living with them, avoidance of misjudgment and husband’s request. It is noted that some of the participants confirmed the positive role of their families in giving hope, providing financial support and consolidating couple’s relationship. This has also been reported in the study conducted by Khodakarami et al. (20).

The findings of the present study showed that the overlap between office hours and treatment schedule with required arrangements for paid and medical leave are among concerns for some infertile couples who hold an office job. This issue jeopardized some patients’ job position as well. The results obtained in India also indicated that many of employers were not much familiar with infertility. Thus, employers need to acquaint themselves with special needs of employees dealing with infertility and arrange the required facilities, such as flexibility in their work schedule in order to attend the treatment programs (43).

Other significant concerns during treatment were enormous treatment expenses and inefficacy of the insurance program for their treatment. Due to financial challenges, some of couples were forced to postpone their treatment or worried about its continuation. In countries where treatment expenses are mainly paid by the patients, financial problems usually play the most effective role in patients’ decision whether to continue or abort their treatment program (44). Most of the couples deal with many financial problems as a part of infertility treatment programs that are not usually covered by insurance agencies. These results are consistent with those obtained by other studies (12, 20, 42). Most of diseases are covered by insurance, but infertility expenses are exceptions to insurance coverage and such discrimination is unfair (43). In this regard, the participated infertile couples requested support from the health care system and governmental authorities and non-governmental agencies. Patients participating in the study conducted by Fahami et al. (12) had similar requests as the participants of current study.

The results obtained in this study showed that spiritual and religious beliefs played an important role in infertile couples’ equanimity and could be considered as a source of support for their adaptation with infertility stress. The results obtained by other studies also indicated that religious beliefs effectively decreased the stress of infertile couples (12, 38).

The results of the present study also showed that information support is considered as another requirement of infertile couples. This has also been reported in the study conducted by Akizuki and Kai (32). Evidence has shown that healthcare providers sometimes tend to underestimate and to ignore a patient’s need for information acquisition (45). Inefficiencies in providing information force patients to obtain information from other sources such as Internet, books or other patients. While providing adequate information to patients by the medical team is considered as patients’ natural right, it ultimately results in gaining patients’ trust and satisfaction and reducing stress of infertile couple (12).

Another finding of the present study was the necessity of educating society and attempting to raise the awareness about infertility and the new treatment methods. One of the main reasons why people surrounding an infertile couple do not know how to treat them is due to their unawareness about different aspects of infertility (40). Educating the society and infertile couples’ families can reduce the psychological burden of these couples (20).

There are some strong points in the current study. Firstly, it studied both infertile men and women and did not focus solely on infertile women. In comparison with individual interviews, we used a dyadic approach and conducted interviews with husband and wife in a joint session that is of great value in infertility-related studies as infertility is a dyadic issue and not an individual one (30). Secondly, all interviews were conducted by an interviewer (the first author), while analysis was carried out by all four researchers, adding to the credibility of the research findings (46). Thirdly, to attain more comprehensive information on infertile couples’ needs, several key informants, such as gynecologists and midwives who had experience with infertile couples, were also interviewed.

A few limitations need to be mentioned. First, as a dyadic approach was used in the interviews with participants, identification of gender differences with regard to the needs experienced by infertile men and women was not possible. Despite the fact that many findings indicate that women, in comparison with their husbands, bear greater negative impacts due to infertility (30), more studies are required to identify infertile men and women’s needs, individually, by conducting dyadic and individual interviews with infertile couples. Second, due to the qualitative nature of the present study, purposive sampling of participants and the limited number of participants, the results of this study cannot be applied to all Iranian infertile couples. Further studies including a larger number of participants categorized in terms of their gender, age, type of infertility, different stages of treatment and different outcomes of treatment are recommended to assess the needs of these patients as per above criteria.

## Conclusion

This study described the four main categories of infertile couples’ needs, part of their challenges and concerns and necessity for cooperative assistance and support. Considering the complete descriptions provided on infertile couples and their needs, the health care professionals and authorities ought to attempt to provide support and consultative programs suiting the infertile couples’ needs. Also it is required to encourage the quality improvement of the healthcare services by development of patientcentered approaches and couple-based interventions so as to reduce infertile patients’ psychological stress induced by fertility problems.
